# Surface Coverage as an Important Parameter for Predicting Selectivity Trends in Electrochemical CO_2_ Reduction

**DOI:** 10.1021/acs.jpcc.2c00520

**Published:** 2022-07-13

**Authors:** Andrew R. T. Morrison, Mahinder Ramdin, Leo J. P. van der Broeke, Wiebren de Jong, Thijs J. H. Vlugt, Ruud Kortlever

**Affiliations:** † Large-Scale Energy Storage, Process & Energy Department, Faculty of Mechanical, Maritime and Materials Engineering, Delft University of Technology, Leeghwaterstraat 39, 2628 CB Delft, The Netherlands; ‡ Engineering Thermodynamics, Process & Energy Department, Faculty of Mechanical, Maritime and Materials Engineering, Delft University of Technology, Leeghwaterstraat 39, 2628 CB Delft, The Netherlands

## Abstract

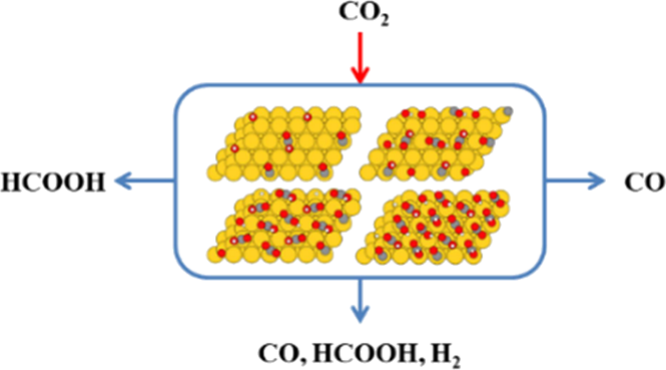

The electrochemical CO_2_ reduction reaction (CO_2_RR) is important for a sustainable future. Key insights into the reaction pathways have been obtained by density functional theory (DFT) analysis, but so far, DFT has been unable to give an overall understanding of selectivity trends without important caveats. We show that an unconsidered parameter in DFT models of electrocatalysts—the surface coverage of reacting species—is crucial for understanding the CO_2_RR selectivities for different surfaces. Surface coverage is a parameter that must be assumed in most DFT studies of CO_2_RR electrocatalysts, but so far, only the coverage of nonreacting adsorbates has been treated. Explicitly treating the surface coverage of reacting adsorbates allows for an investigation that can more closely mimic operating conditions. Furthermore, and of more immediate importance, the use of surface coverage-dependent adsorption energies allows for the extraction of ratios of adsorption energies of CO_2_RR intermediates (COOH_ads_ and HCOO_ads_) that are shown to be predictive of selectivity and are not susceptible to systematic errors. This approach allows for categorization of the selectivity of several monometallic catalysts (Pt, Pd, Au, Ag, Zn, Cu, Rh, W, Pb, Sn, In, Cd, and Tl), even problematic ones such as Ag or Zn, and does so by only considering the adsorption energies of known intermediates. The selectivity of the further reduction of COOH_ads_ can now be explained by a preference for Tafel or Heyrovsky reactions, recontextualizing the nature of selectivity of some catalysts. In summary, this work resolves differences between DFT and experimental studies of the CO_2_RR and underlines the importance of surface coverage.

## Introduction

Transitioning away from carbon-emitting technologies and systems is a crucial part of solving global climate change, but there are several problems that must be solved. The importance of intermittent energy sources in the transition and the probable continued reliance on carbon-emitting devices are two such problems. The electrochemical CO_2_ reduction reaction (CO_2_RR) to commodity chemicals and fuels is can solve these problems by closing the carbon cycle and storing renewable energy in chemical bonds.^1−3^ This reaction can synthesize several C_2+_ chemicals via multielectron reaction pathways, mainly using Cu-based catalysts.^4−6^ Moreover, the two-electron reduction products carbon monoxide and formate/formic acid are of interest as well since there are several sustainable use cases for both. The case for carbon monoxide involves the further processing to produce other chemicals,^3,7,8^ and the case for formate/formic acid is based on the current market for the chemical and future ideas for a bioeconomy or as a liquid reservoir of H_2_ or CO.^9−11^ With these multifaceted applications for the CO_2_RR, understanding the trends in the electrocatalytic performance of different electrode materials toward the CO_2_RR is crucial for catalyst development and optimization for the reaction.^1,12^ Initial experimental studies by Hori et al. mapped the activity and selectivity of many monometallic electrocatalysts for the CO_2_RR.^13^ Development of density functional theory (DFT) models to resolve the observed trends and predict more selective and active catalytic materials is a logical method since selectivity is largely based on the interaction of the electrode surface and adsorbed intermediaries, which DFT is perfectly suited to analyze.^14^

The CO_2_RR is understood to proceed through two possible adsorbed intermediates in an early stage of the reaction: COOH_ads_ and HCOO_ads_. COOH_ads_ reacts to form both formate and CO, while HCOO_ads_ only forms formate, as evidenced by both experimental and theoretical studies.^15−18^ Along with the side production of H_2_ from the hydrogen evolution reaction, these are all the two-electron reactions relevant to the CO_2_RR (see eqs 1^2^,3):^13^
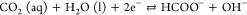
1
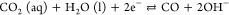
2
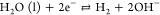
3

Other products, such as hydrocarbons and alcohols, are produced through further reduction of adsorbed CO.^15^ There are other possible adsorbed intermediates, including CO_2_^–^_ads_, the adsorbed products, and of course the intermediates involved in the multielectron reduction toward hydrocarbons and alcohols (e.g., COH_ads_).^15^ However, the current understanding of the selectivity for two-electron reduction products is that materials that bind HCOO_ads_ more strongly than COOH_ads_ produce formic acid, materials with the reverse tend to produce CO, and materials that bind H_ads_ strongly tend to have side reactions,^15−18^ making these species determining in selectivity. However, this basic understanding is yet to match model-based first-principles descriptions, so there are essential details yet to be understood.

Despite the consensus on the two-electron reaction mechanism and rules for product selectivity, designing a DFT model that is able to classify (i.e., predict the major product(s) of the catalyst based on DFT results) the electrocatalytic performance of materials for the CO_2_RR in a holistic manner, across multiple materials as opposed to focusing on a single material, has proven difficult. Studies that elucidate specific mechanisms or are about specific materials are useful, but interpretation remains challenging without a better holistic understanding. Holistic approaches to classify CO_2_RR catalysts have been mostly based on energies of adsorption^17,19−22^ (or quantities similar to adsorption energy, like computational hydrogen electrode potentials^19^). Trends across a small number of surfaces between the key adsorbates and their associated products may be observed, but this is not a proper classification.^17^ However, using the energies of the known intermediates leads to a misclassification of materials.^19^ For example, Ag is predicted to produce formate, but experimental results show a high selectivity toward CO,^23,24^ and Zn is predicted to produce H_2_ or HCOOH but mainly produces CO.^24,25^ Studies that have successfully categorized CO_2_RR catalysts have done so not via the known intermediaries but by using the adsorption energies of H_ads_,^20^ CO_ads_,^21,22^ or OH_ads_.^22^ It is not obvious why these adsorbates should primarily be predictive for CO_2_RR selectivity. In summary, the more holistic DFT models that exist, while generally supporting the current understanding of the two-electron mechanism, all have specific shortcomings. Either they are not able to fully agree with the common understanding of selectivity or they do, but the model in question centers adsorbates that are not otherwise understood to be as important as the model indicates. This difference matters for studies that are trying to interpret finer points of CO_2_RR since it establishes the foundation that those further explanations are based on. The difficulty of classifying CO_2_RR catalysts is also one reason why this study focuses on classification rather than on a more sophisticated prediction—like partial current densities. Some studies have found quantitative relationships with partial current density and DFT models,^17^ but the experimental side of these studies is fragile because exact faradaic efficiencies measured in experiments are dependent on reaction conditions such as the local pH^4,26,27^ and cationic and anionic species^26,28,29^ in the electrolyte, and the mass transfer limitations dependent on the particular cell configuration.^30,31^ Therefore, we focus on accurately predicting the overall selectivity trends.

Of the anomalies mentioned above, the case of Ag has attracted the most interest, likely because Ag is potentially an industrially relevant CO_2_RR catalyst.^32−34^ It has been hypothesized that the predicted preferentially adsorbed HCOO_ads_ on Ag is actually a key component to stabilize the COOH_ads_ intermediary, necessary for CO production, and inhibits hydrogen adsorption.^35^ Another study explains why Ag produces mainly CO on the basis of a difference between mono- and bidentate adsorption of HCOO_ads_.^36^ An alternate approach is to consider different reaction pathways for determining the selectivity.^22^ Alternatively, good agreement can be had with experiments by simply assuming that Ag adsorbs mainly COOH_ads_ (contrary to other DFT results) and further modeling with a multiscale DFT/microkinetic model^37,38^ or with grand-canonical DFT (which allows for a potential-dependent investigation).^39^ In principle, there is nothing stopping these investigations from considering other materials to compare with Ag and thus strengthen their conclusions. In practice, researchers choose not to allocate computational resources toward this. Explaining why non-intermediary adsorbates could be important to selectivity is the other main point of investigation into the anomalies of the holistic DFT. The approach in those studies is often modeling the coverage of nonreacting adsorbates that are thought to be important.^35,40−43^ In fact, such non-intermediary adsorbate studies are the only place in the literature where surface coverage is explicitly considered. Certainly, insights on the CO_2_RR process and possible effects of nonreacting adsorbates are gained, but the effect of the surface coverage on the more holistic adsorption-energy-only work (i.e., coverage of COOH_ads_ and HCOO_ads_) has never been examined for the CO_2_RR to the authors’ best knowledge. The first species considered in a DFT surface coverage investigation should be the species that can determine their selectivity—the key reacting intermediates of COOH_ads_ and HCOO_ads_. This is even more apparent when one considers that COOH_ads_ and HCOO_ads_ are the species that every DFT study must assume a specific coverage of to do DFT work on the CO_2_RR (typically, for fractions between 1/9 and 1/4 for studies of CO_2_RR). Yet, the effect of this parameter has never been investigated. Furthermore, the configuration of adsorbates is not always optimized,^35,41,43^ which can produce errors due to the large configuration space for multiple direct adsorbates.^44^

Here, we leave aside the elucidation of the exact reaction mechanisms on specific materials or in the presence of certain bystander species and focus on improving general DFT models for the CO_2_RR. To improve holistic modeling, we first hypothesize that the selectivity of the CO_2_RR can be predicted based on only the adsorption energies of known adsorbed intermediates. Second, to resolve the issues adsorption-energy-only approaches have had in the past, we hypothesize that the effects of the surface coverage of the known reacting intermediates must be considered. The method employed here is to model several metal surfaces at different surface coverages of COOH_ads_, HCOO_ads_, and H_ads_. The considered surfaces are Pt, Pd, Au, Ag, Zn, Cu, Rh, W, Pb, Sn, In, Cd, and Tl, chosen to give a good selection of different classes of CO_2_RR catalysts. The considered coverages will be from low values in the typical range to high coverages (specifically: 1/6 to 4/6) while keeping the DFT cell size the same to maintain comparability. It would be interesting to examine lower coverages to determine if there is a point where long-range lateral effects fully drop off, but this presents a large computational effort while also maintaining a consistent cell size, so these are not considered for this reason. High coverages are, of course, interesting because they are likely to exist on real catalysts due to specific catalytic effects^6^ or reactor conditions such as high current densities or applied overpotentials,^45^ which will likely be required due to the target current density of 200–1000 mA/cm^2^.^2,46^ They are indicated to exist by, for example, high-pressure CO_2_RR experiments where a suppression of hydrogen evolution is observed^5,47,48^ and changes in CO_2_RR product selectivity due to proton availability^49^ and CO_2_ availability.^50^

High surface coverages have never been directly observed, but the primary operando techniques that are capable of observing CO_2_RR intermediates struggle to quantify coverages, so this should not be surprising in itself. Ultimately, if the hypothesis is correct, the exact range of coverage studied will not be significant as long as it is wide enough to see important trends. Indeed, this work might equally be interesting if the surface coverage is much lower than generally studied, if trends continue below studied coverages.

## Methods

Adsorption energies of COOH_ads_, HCOO_ads_, and H_ads_ are studied for 1, 2, 3, or 4 adsorbates of the same species on 2 × 3 metal surfaces. Energies of adsorption correspond to the following reactions:
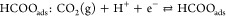

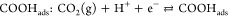

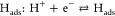


Plane-wave DFT was used to simulate the electrocatalyst surface via use of the Vienna Ab initio Simulation Package (VASP), version 5.2.^51^ The simulations were run via Python scripts using the atomic simulation environment (ASE) library.^52^ The configuration space for multiple adsorbates is very high, even for two adsorbates,^44^ which means it is very difficult to find the configuration of adsorbates that corresponds to the energy minima. In this work, the search for the best configuration was accomplished by the minima hopping algorithm implemented in the ASE^44,52^ that preserves adsorbate identity. It was used to explore several different configurations of adsorbates. Starting configurations were set by researcher intuition, in general, by placing the adsorbates in an “on-top” position (for HCOO_ads_, the two oxygen atoms were both positioned above a surface atom, if possible) and then adjusting the orientation and positions to give a large distance between the atoms of different adsorbates. The algorithm generally found a diverse selection of possible configurations, and alternate starting configurations did not find either new types or lower energies, leading to confidence that the algorithm is exploring the space well. This follows the process in other studies using the same minima hopping algorithm to search for global energy minima for multiadsorbate systems.^42,53^ It should be noted that this procedure comes with the same caveat found in those studies that there is no guarantee that the minima found are the global minima (this is an unavoidable limitation of any global optimization outside of special cases). The initial temperature for the molecular dynamics portion of minima hopping was 2000 K, with an initial acceptance energy of 0.25 eV and a time step of 1 fs.^44^

The ASE minimum hopping algorithm was slightly modified by extending the Hookean constraints, which act to preserve adsorbate identity to allow repulsive Hookean forces. The Hookean constraints in ASE prevent an adsorbate from breaking apart during the molecular dynamics portion of the simulation, holding it together with Hookean forces.^44^ In the algorithm as implemented in the most recent version of the ASE, two atoms constrained can move freely unless the distance between them is larger than a certain threshold and then they are pulled together. In the extension implemented here, two atoms can move freely unless the distance between them is less than a threshold, in which case they are pushed apart. Thus, the extension uses the same type of spring force, but in reverse, to prevent two adsorbates from combining and therefore losing their identities in that way.

**Figure 1:**
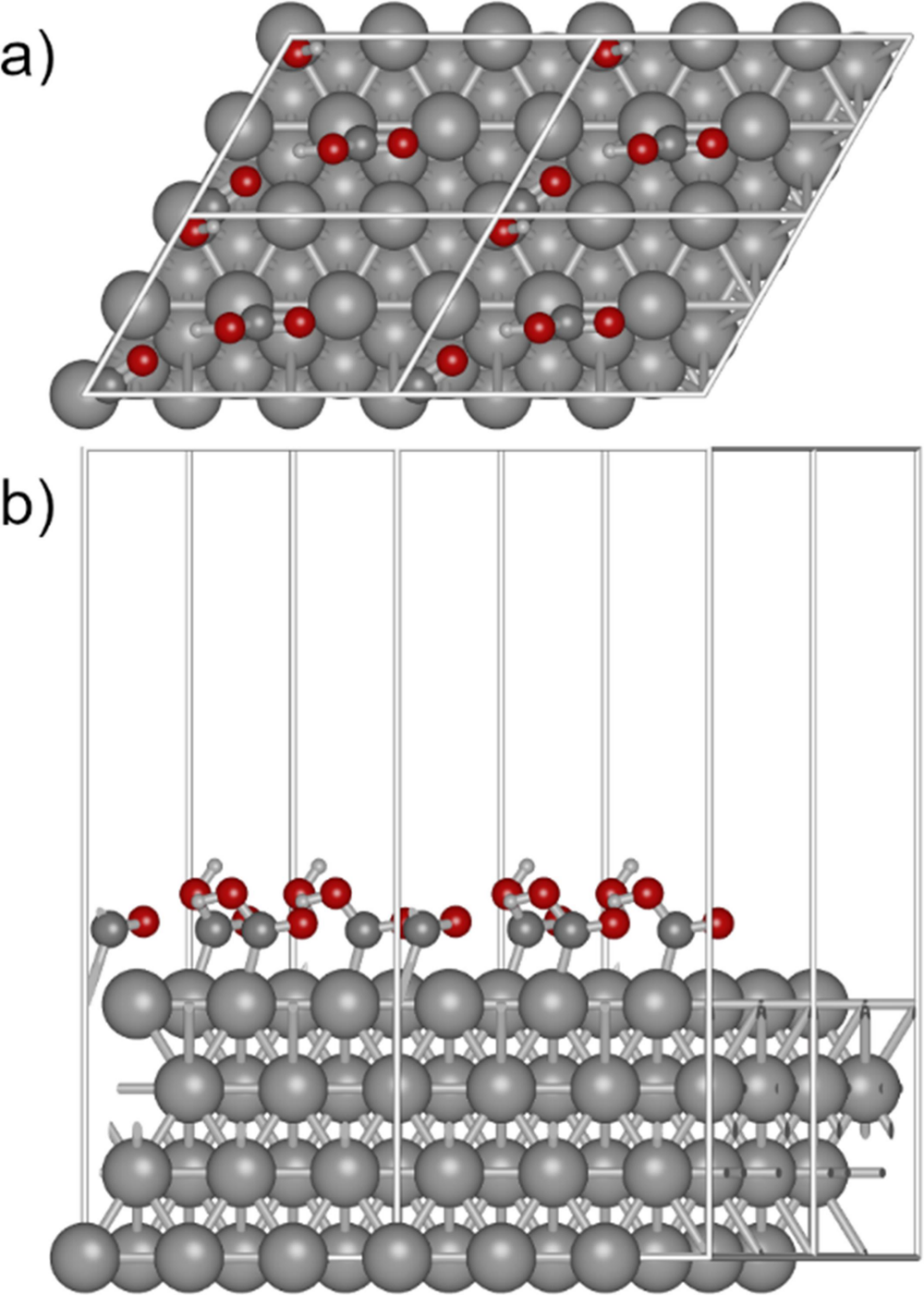
Ag 2 × 3 cell with 2 COOH_ads_ repeated once in both planar directions viewed from the (a) top and (b) side.

The VASP simulations handled exchange and correlation with the BEEF-vdW functionals.^54−56^ These have been shown to be accurate for surface adsorption calculations compared with functionals such as PW91, HSE, or RPBE and comparable to functionals, which also describe vdW interactions.^57−68^ BEEF-vdW has also been used in a number of studies on CO_2_RR,^19,20,22,35,38,43^ indicating that it is a good functional for the reactions. The ionic cores were described by a plane-wave basis set employing the Vanderbilt ultrasoft pseudopotential (US-PP).^69^ The Brillion zone was sampled using a 4 × 4 × 1 Monkhorst–Pack grid of *k*-points.^70^ A kinetic energy cutoff of 500 eV and a density energy cutoff of 5000 eV were used. The system modeled was a 2 × 3 × 4 supercell with at least 16 Å of vacuum added in the direction perpendicular to the surface. The crystal structure of the surface is FCC for Pd, Pt, Rh, Au, Cu, Ag, and Pb; HCP for Cd, Tl, and Zn; BCC for W; and tetragonal for Sn (space group 141) and In (space group 139) with optimized lattice parameters. Figure 1 shows an example of the supercell with 2 COOH_ads_. The top two layers of the metal were allowed to relax and the bottom two held in place, except in minima hopping calculations the top layers were also locked (this is known to cause a minimal error and represents significant computation saving for these calculations^44^). The convergence criterion for the energy optimization was a maximum force of 0.02 eV/A per atom. Energies to account for zero-point energy according to the values of Chan et al.,^71^ and further corrections were made to gas-phase H_2_ and to COOH_ads_, 0.09 and 0.25 eV.^19,72^ These details match closely with the work of Yoo et al., which was for a similar system, and the methods here were benchmarked via values in the Supporting Information of that article.^19^

## Results and Discussion

**Figure 2:**
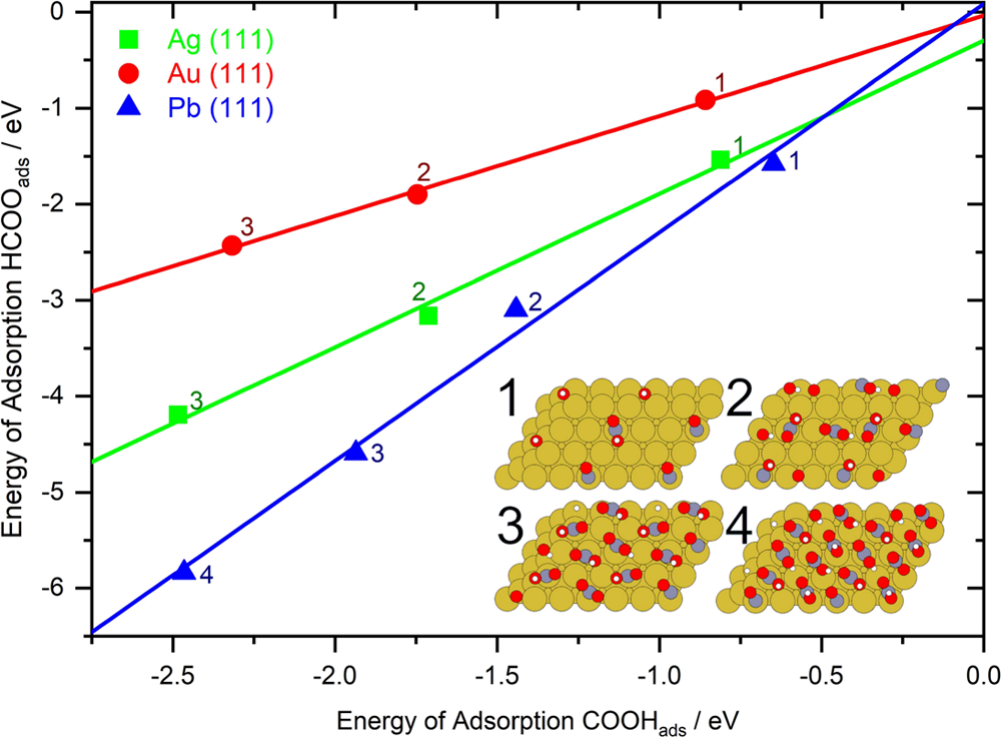
Typical plots of *E*_ads_ COOH_ads_ vs *E*_ads_ HCOO_ads_ for 1–4 adsorbates of the same species in a 2 × 3 section of a Ag (green), Au (red), and Pb (blue) surface. Each point is labeled with the number of adsorbates on a 2 × 3 surface cell it represents. The lines are plotted from linear regressions of at least 3 of the points—discounting the 4th point if the surface with 4 adsorbates was too packed. Inset shows 1–4 COOH_ads_ in a 2 × 3 Au cell repeated twice in each direction as an example. See Section S2 in the Supporting Information for this type of plot for other metals.

In Figure 2, the total energies of adsorption for *n* COOH_ads_ and *n* HCOO_ads_ are plotted against each other for all *n* less than or equal to the number of adsorbates that fit in a 2 × 3 surface cell of Au, Ag, and Pb (the surface can become too packed to easily add adsorbates—see Figure 2 inset).

**Figure 3:**
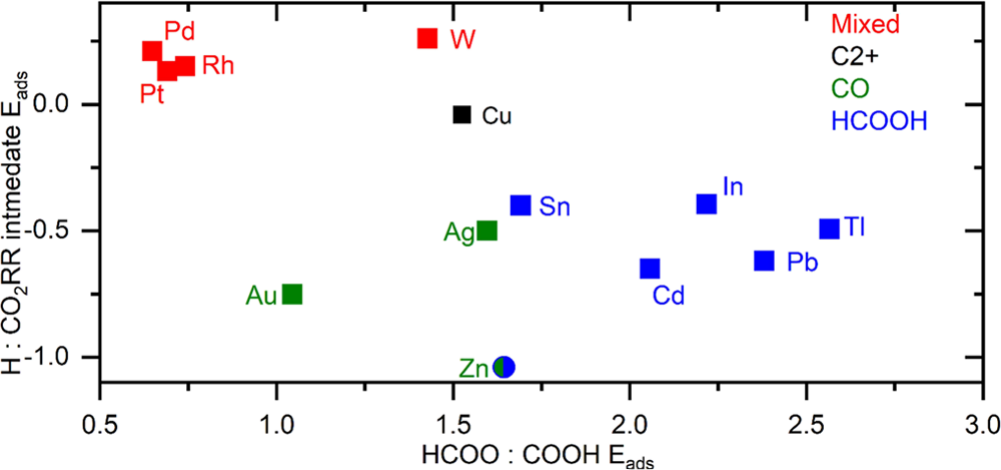
Summary of the data in Tables 1, 2, and S1 (Supporting Information). The ratio between the adsorption energy of H_ads_ and the most stable CO_2_RR intermediate (Tables 2 and S1) is plotted vs the ratio between the energy of adsorption of HCOO_ads_ and COOH_ads_ (Table 1). It is clearly seen here how the different classes of CO_2_RR catalysts group themselves.

These three materials are representative of materials studied here (see Section S2 in the Supporting Information). The parameters of the fits for all the considered metals can be seen in Table 1 for COOH_ads_ vs HCOO_ads_, Table 2 for COOH_ads_ vs H_ads_, and Table S1 in the Supporting Information for HCOO_ads_ vs H_ads_ (and all summarized in Figure 3). The total energy of adsorption increases as *n* increases, which is expected as doubling *n* will require approximately twice the adsorption energy, disregarding other effects. As can be seen in Figure 2, the energies of adsorption increase proportionally by the same amount for both HCOO_ads_ and COOH_ads_, up to 3 or 4 adsorbates (this is surface-dependent, see Section S2 in the Supporting Information). The uneven spacing of points along the lines indicates that there are adsorbate–adsorbate interactions, but the linear relationship in this space indicates that these interactions are approximately the same for COOH_ads_ as for HCOO_ads_ since if interadsorbate interactions differed between the two, the data would not be linear but would curve toward the adsorbate more stabilized by higher coverages (or less destabilized). This is interesting partially because for COOH_ads_, hydrogen bonding could be expected to stabilize the adsorbates at high surface coverage, whereas that is not possible for HCOO_ads_. Indeed, in several of the optimized geometries, the hydrogen atom in COOH_ads_ takes up a position to the side of the oxygen atom rather than on top, indicating hydrogen bonding. Whether an adsorbed structure displays hydrogen bonding does depend on surface coverage (see Section S4 in the Supporting Information). However, the greatest number of hydrogen-bonded structures is actually found at medium surface coverages. A likely explanation for this is that the stabilizing effect of hydrogen bonding competes with the destabilizing effect of an increase in the footprint of the molecule on the surface, which the H bonding configuration requires. The larger footprint will make it more difficult to fit all the COOH_ads_ in their optimum positions and also create more repulsion for nearby adsorbates not participating in the bond. This explains why the presence of hydrogen-bonded COOH_ads_ does not curve the plots in Figure 2.

**Table 1:** Parameters of the Linear Fit for Plots of the Total Energy of Adsorption for the HCOO and COOH Intermediaries Plotted against Each Other for 1–4 Adsorbates of the Same Type of Adsorbate in a 2 × 3 Section of the Surface as Calculated by DFT alongside the Main Products Seen in Experimental Investigation in the Literature for That Surface^a^

surface	slope (HCOO_ads_:COOH_ads_*E*_ads_ ratio)	intercept/eV	main experimental products (5 mA·cm^–2^)^24^	main experimental products (200 mA·cm^–2^)^73^
Pd (111)	0.65	–0.39	H_2_, CO	HCOOH, CO, H_2_
Pt (111)	0.69	–0.10	H_2_	H_2_, HCOOH
Rh (111)	0.74	–0.60	no data	CO, HCOOH, H_2_
Au (111)	1.04	–0.04	CO	CO
W (110)	1.43	0.95	no data	H_2_, HCOOH
Cu (111)	1.52	0.00	CO, C2, and up	CO, C2, and up
Ag (111)	1.60	–0.30	CO	CO
Zn (0001)	1.64	0.68	CO	CO, HCOOH
Sn (100)	1.69	0.02	HCOOH	HCOOH
Cd (0001)	2.06	–0.13	HCOOH	no data
In (001)	2.22	0.12	HCOOH	HCOOH
Pb (111)	2.38	0.09	HCOOH	HCOOH
Tl (0001)	2.57	0.15	HCOOH	no data

a Examples of these plots can be seen in Figure 2. The table is sorted by a slope, with the main products noted for 5 mA·cm^–2^^24^ and 200 mA·cm^–2^^73^ (categorized according to the experimental study from the literature). See the Supporting Information material classification (Section S1) and further plots (Section S2).

**Table 2:** Parameters of the Linear Fit of Total Energy of Adsorption for the H_ads_ and COOH_ads_ Intermediaries vs Each Other for 1–4 Adsorbates of the Same Type of Adsorbate in a 2 × 3 Section of the Surface as Calculated by DFT^a^

surface	slope (H_ads_:COOH_ads_*E*_ads_ ratio)	intercept/eV	main experimental products (5 mA·cm^–2^)^24^	main experimental products (200 mA·cm^–2^)^73^
W (110)	0.26	0.08	no data	H_2_, HCOOH
Pd (111)	0.21	0.03	CO, H_2_	HCOOH, CO, H_2_
Rh (111)	0.15	0.01	no data	CO, HCOOH, H_2_
Pt (111)	0.13	0.02	H_2_	H_2_, HCOOH
Cu (111)	–0.04	–0.02	CO, C_2_, and up	CO, C_2_, and up
Ag (111)	–0.50	–0.01	CO	CO
Sn (100)	–0.68	–0.20	HCOOH	HCOOH
Au (111)	–0.75	–0.21	CO	CO
In (001)	–0.98	–0.15	HCOOH	HCOOH
Zn (0001)	–1.04	0.04	CO	CO, HCOOH
Tl (0001)	–1.27	–0.23	HCOOH	no data
Cd (0001)	–1.33	–0.01	HCOOH	no data
Pb (111)	–1.47	–0.25	HCOOH	HCOOH

a The table is sorted by a slope, with the main products noted for 5 mA·cm^–2^ and 200 mA. See the Supporting Information material classification (S1) and further plots (S2).

Of course, a line has two properties commonly called the slope and the intercept. The meaning of these two properties for the linear regressions and how they can explain/predict the CO_2_RR selectivity of materials is discussed here.

As can be seen in Figure 2, the fit for Ag has a significant nonzero intercept, and for Pb, it may be slightly out as well. In fact, this is typical of the linear regressions for this data. The intercepts of these fits should all be 0, since at 0 adsorbates, there should be 0 adsorption energy for both species. A nonzero intercept indicates that there is a systematic bias (i.e., the same size of effect regardless of the number of adsorbates) that influences calculated adsorption energies. The nonzero intercepts begin to show the value of this technique since it is a bias that would be present in normal DFT calculations, but there would be no way to see it since only a single point is calculated for each surface. The fact that for some of the surfaces the magnitude is tens of millielectronvolt means that the systematic bias would be enough to influence predictions, if they were based on a single point. The possible sources of the nonzero intercepts can be divided into three categories: biases to do with the surface energy, to do with the adsorbate-surface energy, or to do with the lateral interaction of adsorbates. The first possibility can be eliminated because if it were from the surface energy, all three fits (two in Tables 1 and 2 and the third in Section S2 of the Supporting Information) for a given material would have the same nonzero intercepts, which is not the case. The second can likely be eliminated since this would not be systematic since it would be multiplied by the number of adsorbates. Thus, these nonzero intercepts must arise from adsorbate–adsorbate interactions. This should also scale with the number of adsorbates but not necessarily linearly like with surface-adsorbate energies. Within lateral interactions, either there is an error in the DFT code or configuration arising from or dealing with lateral interactions (even effecting cells with one adsorbate because of the periodic boundary conditions) or there are long-range lateral interactions that fall away at very low surface coverages. As has already been mentioned, studying very low coverages is computationally intensive and is not done in the present study. Whichever way it is, these nonzero intercepts show that studying coverage-dependent adsorption energies is important. Furthermore, a solution to the effect of nonzero intercepts is contained in the other parameter of the linear regressions of the DFT data: the slope.

The slope is actually where the linear relationship between the HCOO_ads_ and COOH_ads_ adsorption energies has utility because the slope is necessarily the ratio of the two adsorption energies (see Section S3 in the Supporting Information for more details). The ratio is an interesting quantity since it is a single number that can be used to compare the relative preference of a surface between two adsorbates. In this case, a higher ratio indicates that HCOO_ads_ is more strongly adsorbed than COOH_ads_ and vice versa for a lower ratio. Calculating the ratio from the linear regression of coverage-dependent adsorption energies rather than from adsorption energies at a single coverage is superior because it eliminates the effects of the nonzero intercepts described above while secondarily reducing random errors. The slopes of coverage-dependent adsorption energy can thus be examined as a descriptor to predict if the surface mainly produces CO or HCOOH. In Table 1, the slopes and intercepts of fits for several different metal surfaces are shown, and the main product(s) observed during experimental studies are listed. The data in Table 1 shows that the slope can be used to sort the materials with a single primary product by their selectivities. All surfaces with a fitted slope larger than 1.65 primarily produce formic acid/formate (HCOOH), and surfaces with a fitted slope less than 1.65 will primarily produce CO. This is consistent with the understanding of the two intermediates. Surfaces where HCOO_ads_ is more strongly adsorbed (high slope) produce mainly HCOOH since HCOO_ads_ reduces only to HCOOH, while surfaces where the slope is low adsorb more COOH_ads_ that reduces to either CO or HCOOH. The sorting of Cu as a CO-producing material by the HCOO_ads_:COOH_ads_ ratio is consistent with the understanding that hydrocarbon products typically produced on Cu surfaces are known to result from the further reduction of adsorbed CO.^15^ It is especially of note that this method can place Ag and Zn on the CO-producing side of the divide. Also, Zn being at the boundary is consistent with work, showing that its main product is flexible.^74,75^ Another interesting point is Sn, being near the boundary on the HCOOH side. This is consistent with several studies that have shown that Sn can be modified or added to synthesize a catalyst that either produces CO^76−79^ or HCOOH.^78−80^

Another important distinction for two-electron products of the CO_2_RR is the difference between surfaces that predominantly produce a single product versus those that produce multiple products. As can be seen in Table 1, the multiple product surfaces tend to adsorb COOH_ads_ more preferentially than the CO-selective surfaces, with HCOO_ads_:COOH_ads_ ratios below 1.5 being multiproduct surfaces. However, Au, a catalyst that is selective for CO, shows that this cannot be the whole story.

To determine the properties of a multiproduct CO_2_RR catalyst, the COOH_ads_:H_ads_ ratio will be used instead of the HCOO_ads_:COOH_ads_ adsorption energy ratio (see Table 2). The data in Table 2 is based on the same type of fits as seen in Figure 2, but with H_ads_ adsorption energy substituted for HCOO_ads_ (see Section S2 in the Supporting Information). In Table 2, higher slopes should tend to adsorb H_ads_ more strongly than COOH_ads_ based on the same reasoning as the HCOO_ads_:COOH_ads_ ratio for the difference between CO and HCOOH. As can be seen in Table 2, surfaces that have a higher slope (surfaces that more strongly adsorb hydrogen) do correspond to multiple product materials and those that produce predominately a single product all have a lower slope. Thus, the relative availability of adsorbed hydrogen determines whether surfaces that tend to adsorb COOH_ads_ produce mostly CO or produce multiple products. Finally, the position of Cu, at the boundary between single product and multiple product materials, is consistent with this hypothesis since it reflects the need for a balance of adsorbed hydrogen and CO_2_ reduction intermediaries to produce C_2_ and higher products.^81^

To further put Table 1 and Table 2 in context and offer insights into the underlying mechanisms, some information about the electronic structure and the low coverage adsorption energies can be found in Table S2 in the Supporting Information. The energies of adsorption at low coverage underscore the used technique presented here showing no clear-cut delimitation of the different categories of the catalyst. There is an association between metals with a low d-band center (or the p-block metals) and the overall efficiency at the CO_2_RR; however, the association between that parameter and which CO_2_RR intermediatory they tend to adsorb is much less clear. Another interesting potential association can be seen with the density of surface atoms, also found in Table S2.

**Figure 4:**
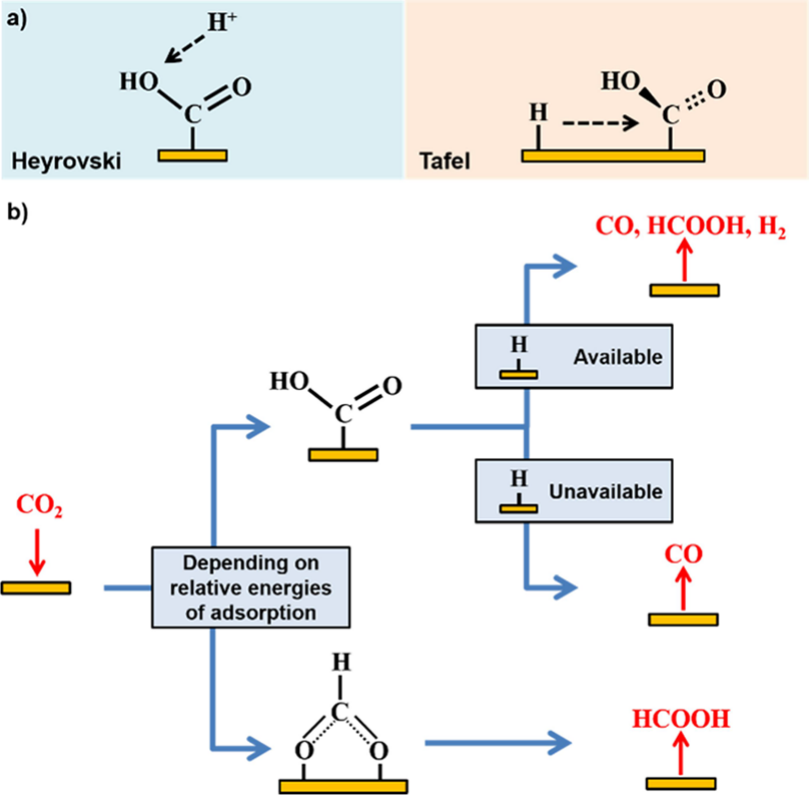
(a) Schematic of the proposed tendency of Heyrovsky vs Tafel mechanism for the CO_2_RR. In the Heyrovsky mechanism, CO is the more likely product because the carbon atom is screened from reacting. In the Tafel mechanism, the angle of reaction is from the side, so either the carbon or OH group is open for the reaction. (b) Schematic of the deciding factors for the different types of the CO_2_RR catalyst.

So far, we have shown that our method can distinguish between catalysts that will produce mainly formate and mainly CO and between surfaces that produce mainly a single product (CO or formate) and those that have a more even distribution of products. The product distribution in the multiproduct surfaces does remain unpredictable, and the prime question here is why these surfaces that, according to the model presented, tend to strongly adsorb COOH_ads_ can produce significant amounts of HCOOH when COOH_ads_ does not react to HCOOH in CO-selective surfaces (e.g., Pd can produce a large amount of HCOOH and Au makes mainly CO, but both tend to prefer the COOH_ads_ intermediate). According to the reasoning until now, multiproduct surfaces should make CO if they are reducing CO_2_ at all, since they all tend to adsorb COOH_ads_. A hypothesis to resolve this will be proposed. If a surface has a large amount of H_ads_ and fewer COOH_ads_ (the conditions for multiproduct surfaces), this condition will favor a Tafel mechanism for further reduction of COOH_ads_. Surfaces that have less adsorbed H_ads_ and more COOH_ads_ (the likely condition of primarily CO-producing surfaces) will favor a Heyrovsky mechanism, which is through a reaction with an aqueous H, for further reduction. The Heyrovsky mechanism will naturally lead to production of CO rather than HCOOH because the upward positioned oxygen atom and hydroxyl group will screen the carbon from the incoming hydrogen. If the hydrogen reacts with the hydroxyl group, it will produce CO. Alternatively, if the reacting hydrogen is adsorbed to the catalyst, it can readily react with either the carbon of the COOH_ads_ or its hydroxyl group. Thus, COOH_ads_ reacting via the Tafel mechanism can readily form either CO or HCOOH. This is in line with the experimental evidence that HCOOH is selected for when H_ads_ is made more available than aqueous H.^82−84^ A schematic representation of this explanation can be seen in Figure 4a along with a summary of a guide to the selectivity of catalysts in Figure 4b. Thus, the selectivity of the multiproduct surfaces should be found by analyzing the further reduction of COOH_ads_, rather than focusing on the adsorption energy of the initial intermediates. This is relevant for Pd selectivity, which can be made to favor either CO or HCOOH, even with catalysts that are entirely or mostly Pd.^85−92^ Some analyses suggest that this can be explained by the difference between the adsorption energy of COOH_ads_ and HCOO_ads_.^85−87^ However, as seen here, the approach of focusing on the adsorption energy of CO^88−91^ and/or the production of HCOOH through the COOH path^92^ should yield more insights since they deal with the further reduction of the COOH_ads_ intermediate, which is more significant.

## Conclusions

We have demonstrated that surface coverage effects are essential for predicting the selectivity of metal catalysts for the CO_2_RR. Specifically, taking coverage-dependent adsorption energies as a descriptor of CO_2_RR catalyst selectivity is necessary to categorize catalysts by which two-electron products they tend to make. This success is due to the ability of surface coverage-dependent adsorption energies to eliminate systematic effects likely associated with adsorbate–adsorbate interactions. Importantly, this method successfully categorizes all the considered metals correctly, including previously problematic ones, and it does this through only adsorbates that are known to participate in the reaction. This combination of results has not been achieved in a previous study, so it is an important result in itself.

Our model supports the story told to explain two-electron product selectivity. That is to say, the difference between HCOOH and CO-producing surfaces is seen to lie in the relative energies of adsorption of COOH_ads_ and HCOO_ads_. The difference between electrodes that produce CO and multiple product surfaces (both categories tending to adsorb COOH_ads_) is the relative energy of adsorption of H_ads_ to COOH_ads_. Furthermore, the reason that surfaces that tend to adsorb COOH_ads_ are able to produce HCOOH in the case of multiproduct surfaces, but this ability is limited in CO-selective surfaces, is proposed to be a difference in Heyrovsky vs Tafel reaction mechanisms for further reduction of the COOH_ads_ intermediate. This overall scheme can be seen in Figure 4b, which highlights how the possibility of COOH_ads_ reducing to HCOOH is significant. In fact, this study supports the two-electron selectivity story more so than other studies. This result is important in more than its own right because it indicates that less special explanations of certain surfaces or mechanisms may be needed than previously thought. Concretely, it can change interpretation of previous results. For example, this can already help to explain the selectivity of materials like Ag or Zn, and it recontextualizes the nature of Pd-based materials. It also indicates how the ability to tune Zn- or Sn-based catalysts for CO and HCOOH, as has been already seen in the literature can be understood. Furthermore, more confidence can be placed on the use of a DFT model that takes surface coverage into account to (re)examine the selectivity of (new) electrocatalytic materials for the CO_2_RR.

## Supporting Information Available

Material classification vs activity, further data and plots, elimination of systematic errors, and hydrogen bonding in COOH_ads_ (PDF)
