# Supplementary material for: Surface Coverage as an Important Parameter for Predicting Selectivity Trends in Electrochemical CO2 Reduction
Source: J Phys Chem C Nanomater Interfaces. 2022 Jul 13;126(29):11927–36. doi: 10.1021/acs.jpcc.2c00520 (PMC9340765; doi:10.1021/acs.jpcc.2c00520)
Supplement: Supplementary file 1 — jp2c00520_si_001.pdf [file jp2c00520_si_001.pdf]

Supplementary Information to:

# Surface Coverage as an Important Parameter for Predicting Selectivity Trends in Electrochemical $CO_2$ Reduction

Dr. Andrew R.T. Morrison[a], Dr. Mahinder Ramdin[a][b], Dr. Leo J. P. van der Broeke[b], Prof. Wiebren de Jong[a], Prof. Thijs J.H. Vlugt[b], Dr. Ruud Kortlever* [a]

[a] Large-Scale Energy Storage, Process & Energy Department,
Faculty of Mechanical, Maritime and Materials Engineering,
Delft University of Technology, Leeghwaterstraat 39, 2628 CB Delft, The Netherlands

[b] Engineering Thermodynamics, Process & Energy Department,
Faculty of Mechanical, Maritime and Materials Engineering,
Delft University of Technology, Leeghwaterstraat 39, 2628 CB Delft, The Netherlands

## Table of Contents

S1. Material Classification vs. Activity ....3

S2. Further data and plots ....4

S3 Eliminate Systematic Error ....9

S4 Hydrogen Bonding in $COOH_{ads}$ ....10

References....11

## S1. Material Classification vs. Activity

A descriptor of the catalytic properties is necessary when modelling the selectivity of the surface – i.e. what the model will be trying to predict. A classic approach is to take the current density. This has had success in the hydrogen evolution [1], but in the $CO_2$RR it must be expanded to consider the partial current density [2]. However, the possible interactions between the multiple surface reactions make it difficult isolate activity in this manner. Another possibility is what is here termed the classification approach where catalysts are sorted into different classes, in the $CO_2$RR case the categories used have been by the predominate product either CO, HCOOH or $H_2$.

The classification approach was followed here. Data from both low current densities (5 mA/cm$^2$) [3], and high current densities (200 mA/cm$^2$, at elevated pressure) [4] have been combined to classify materials. Two types of materials can be identified: those that produce mainly a single $CO_2$ reduction product at both high and low current densities and those that have a wide range of products. The first category is again broken down into CO producing materials and HCOOH producing materials. Interestingly, although it is true there are several materials that produce only $H_2$ at low current densities, at high current densities there are no single product $H_2$ catalysts.

It is obvious the $HCOO_{ads}$-$COOH_{ads}$ slope can be qualitatively predictive from Table 1 in the main text, but could it also have a quantitative value? In Figure S1**Error! Reference source not found.** the slope is plotted vs. the $CO_2$ conversion efficiency toward HCOOH at 200 mA/cm$^2$, defined as the percentage of $CO_2$RR current that is going toward HCOOH, is plotted. As the slope increases for the single product materials, and the surfaces tend to adsorb $HCOO_{ads}$ instead of $COOH_{ads}$, the conversion efficiency increases. It seems it can be quantitative, but this point is not as strong because of a lack of materials that produce only single products to fill this plot out. However, no such relationship can be determined from the partial current density for these materials at 5 mA/cm$^2$. This highlights the difficulty of using a model to quantify selectivity – the uncertainty in how to quantify selectivity leads to a degree of researcher bias.

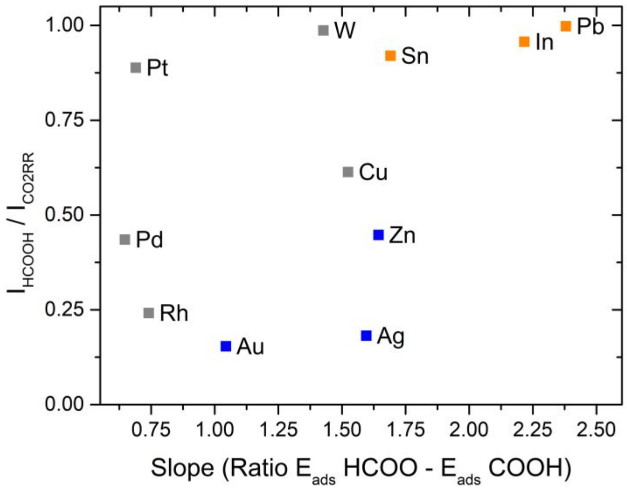


**Figure S1.** The slope, being the ratio between the adsorption energy of the COOH and HCOO intermediaries (see Table 1), vs. the efficiency of converting $CO_2$ to HCOOH at 200 mA [7] (i.e. a faradaic efficiency ignoring $H_2$ production). For those materials that mainly produce CO or HCOOH, the fraction of $CO_2$ that is converted to HCOOH increases as the slope increases.

## S2. Further data and plots

**Table S1:** Parameters of the linear fit for plots the total energy of adsorption for the $H_{ads}$ and $HCOO_{ads}$ intermediaries plotted against each other for 1-4 adsorbate of the same type of adsorbate in a 2x3 section of the surface as calculated by DFT. The table is sorted by slope, with the main products noted for 5 mA/cm$^2$ and 200 mA/cm$^2$.

| Metal | Slope | Intercept | Low Current Product [3] | High Current Product[4] |
|---|---|---|---|---|
| Pd (111) | 0.3066 | 0.1224 | CO, $H_2$ | HCOOH, CO, $H_2$ |
| Rh (111) | 0.1948 | 0.1027 | No data | CO, HCOOH, $H_2$ |
| W (110) | 0.1842 | -0.0955 | No data | $H_2$, HCOOH |
| Pt (111) | 0.1837 | 0.0343 | $H_2$ | $H_2$, HCOOH |
| Cu (111) | -0.0228 | -0.0125 | CO, C2 and up | CO, C2 and up |
| Ag (111) | -0.3104 | -0.0918 | CO | CO |
| In (001) | -0.3948 | 0.0555 | HCOOH | HCOOH |
| Sn (0001) | -0.3998 | -0.1898 | HCOOH | HCOOH |
| Tl (0001) | -0.4931 | -0.1569 | HCOOH | No data |
| Pb (111) | -0.6193 | -0.2017 | HCOOH | HCOOH |
| Zn (0001) | -0.6409 | 0.4882 | CO | CO, HCOOH |
| Cd (0001) | -0.6510 | -0.1010 | HCOOH | No data |
| Au (111) | -0.7094 | -0.2253 | CO | CO |

**Table S2: A modified version of Table 1 from the main text with the addition of information about the electronic structure of the metal surface (the d-band center for transition metals), low coverage adsorption rates, and the DFT cell surface area.**

| Surface | Slope | D-band Center[5-6] / eV | Low coverage Adsorption Energy/ eV | | | Cell area Å$^2$ | Main products [3-4] |
|---|---|---|---|---|---|---|---|
| | | | COOH | HCOO | H | | |
| Pd (111) | 0.65 | -1.83 | -1.71 | -1.42 | -0.33 | 48.12 | HCOOH, CO, $H_2$ |
| Pt (111) | 0.69 | -2.25 | -1.85 | -1.34 | -0.22 | 48.46 | $H_2$, HCOOH |
| Rh (111) | 0.74 | -1.73 | -1.89 | -1.89 | -0.28 | 43.40 | CO, HCOOH, $H_2$ |
| Au (111) | 1.04 | -3.56 | -0.85 | -0.91 | 0.47 | 53.87 | CO |
| W (110) | 1.43 | -1.9 | -2.66 | -3.04 | -0.65 | 52.15 | $H_2$, HCOOH |
| Cu (111) | 1.52 | -2.67 | -1.24 | -1.86 | 0.03 | 41.00 | CO, C2 + |
| Ag (111) | 1.60 | -4.3 | -0.81 | -1.53 | 0.42 | 53.91 | CO |
| Zn (0001) | 1.64 | -3.88 | -0.75 | -0.52 | 0.58 | 40.54 | CO, HCOOH |
| Sn (100) | 1.69 | P-band | -1.06 | -1.73 | 0.82 | 59.20 | HCOOH |
| Cd (0001) | 2.06 | -8.5 | -0.75 | -1.73 | 1.05 | 58.05 | HCOOH |
| In (001) | 2.22 | P-band | -0.83 | -1.71 | 0.75 | 67.17 | HCOOH |
| Pb (111) | 2.38 | P-band | -0.64 | -1.57 | 0.83 | 78.70 | HCOOH |
| Tl (0001) | 2.57 | P-band | -0.79 | -1.90 | 0.80 | 79.10 | HCOOH |

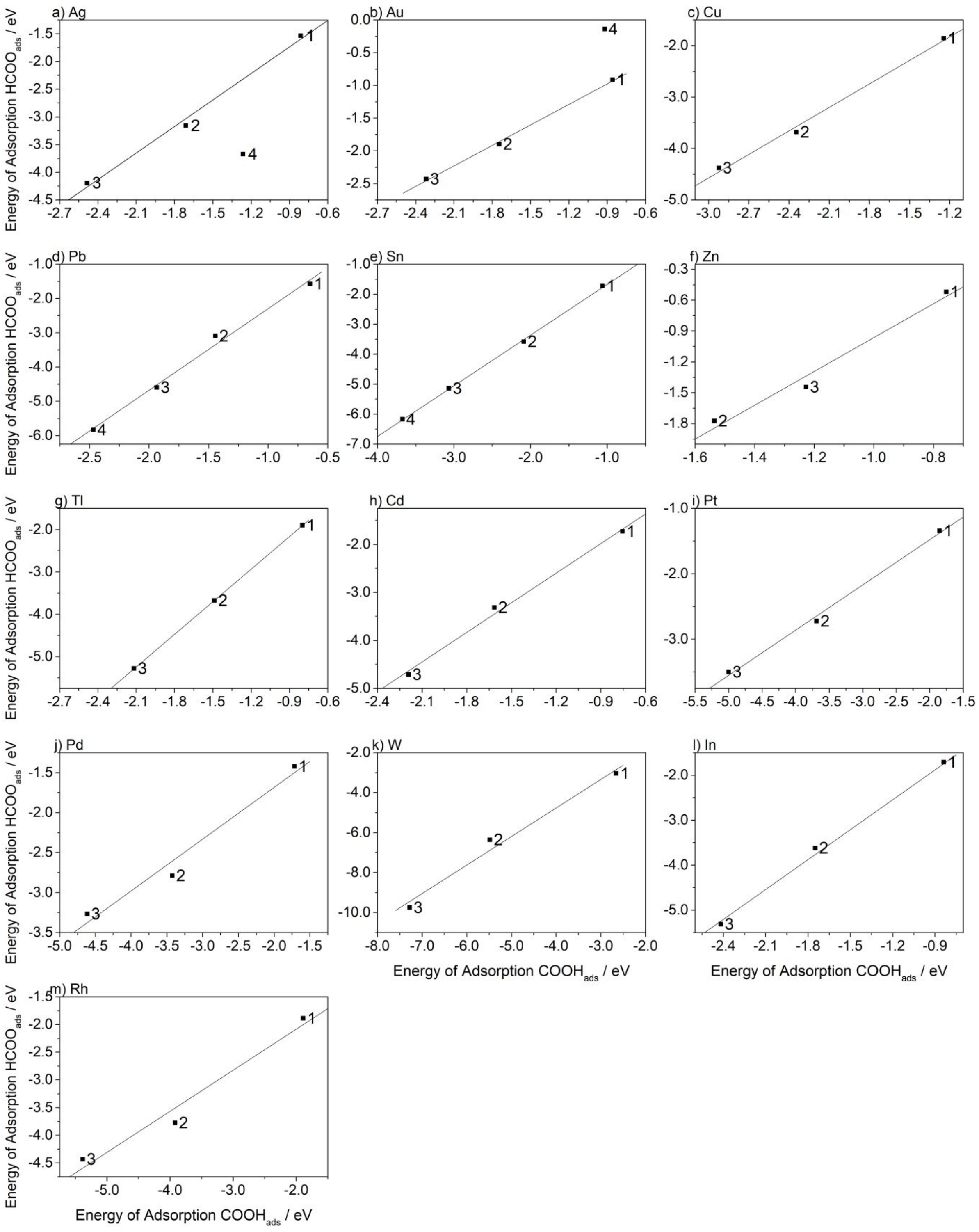


**Figure S2:** All plots of $E_{ads}$ $COOH_{ads}$ vs. $E_{ads}$ $HCOO_{ads}$ for 1-4 adsorbate of the same type of adsorbate in a 2x3 section of each metal as listed in the figure. These are the energies of adsorption of 1-4 $CO_2$ adsorbing through a proton transfer and are calculated with DFT (see section S1 of the for more details).Each point is labelled with the number of adsorbates (either all $COOH_{ads}$ or all $HCOO_{ads}$) on a 2x3 surface cell it represents. The lines are plotted from linear regressions of at least 3 of the points – discounting the 4th point if the surface with 4 adsorbates was too packed for a reasonable measurement. The parameters for the fits can be found in the Table 1 of the main article.

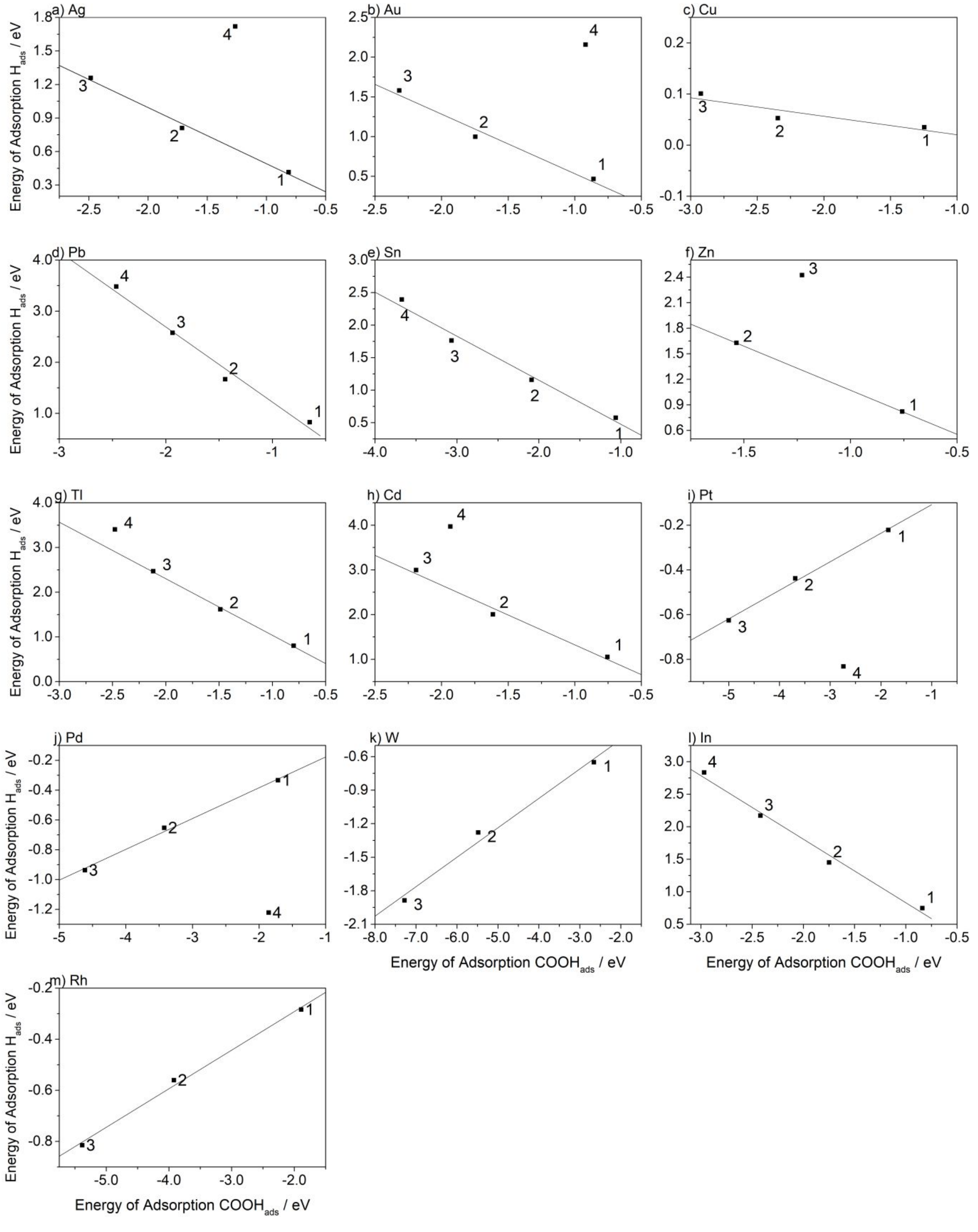


**Figure S3.** All plots of $E_{ads}$ $COOH_{ads}$ vs. $E_{ads}$ $H_{ads}$ for 1-4 adsorbate of the same type of adsorbate in a 2x3 section of each metal as listed in the figure. The former is the energies of adsorption of 1-4 $CO_2$ adsorbing on the surface through a proton transfer while the latter is the energy of adsorption of a proton. Both are calculated with DFT (see section S1 of the for more details). Each point is labelled with the number of adsorbates (either all $COOH_{ads}$ or all $H_{ads}$) on a 2x3 surface cell it represents. The lines are plotted from linear regressions of at least 3 of the points – discounting the 4th point if the surface with 4 adsorbates was too packed for a reasonable measurement. The parameters for the fits can be found in the Table 1 of the main article.

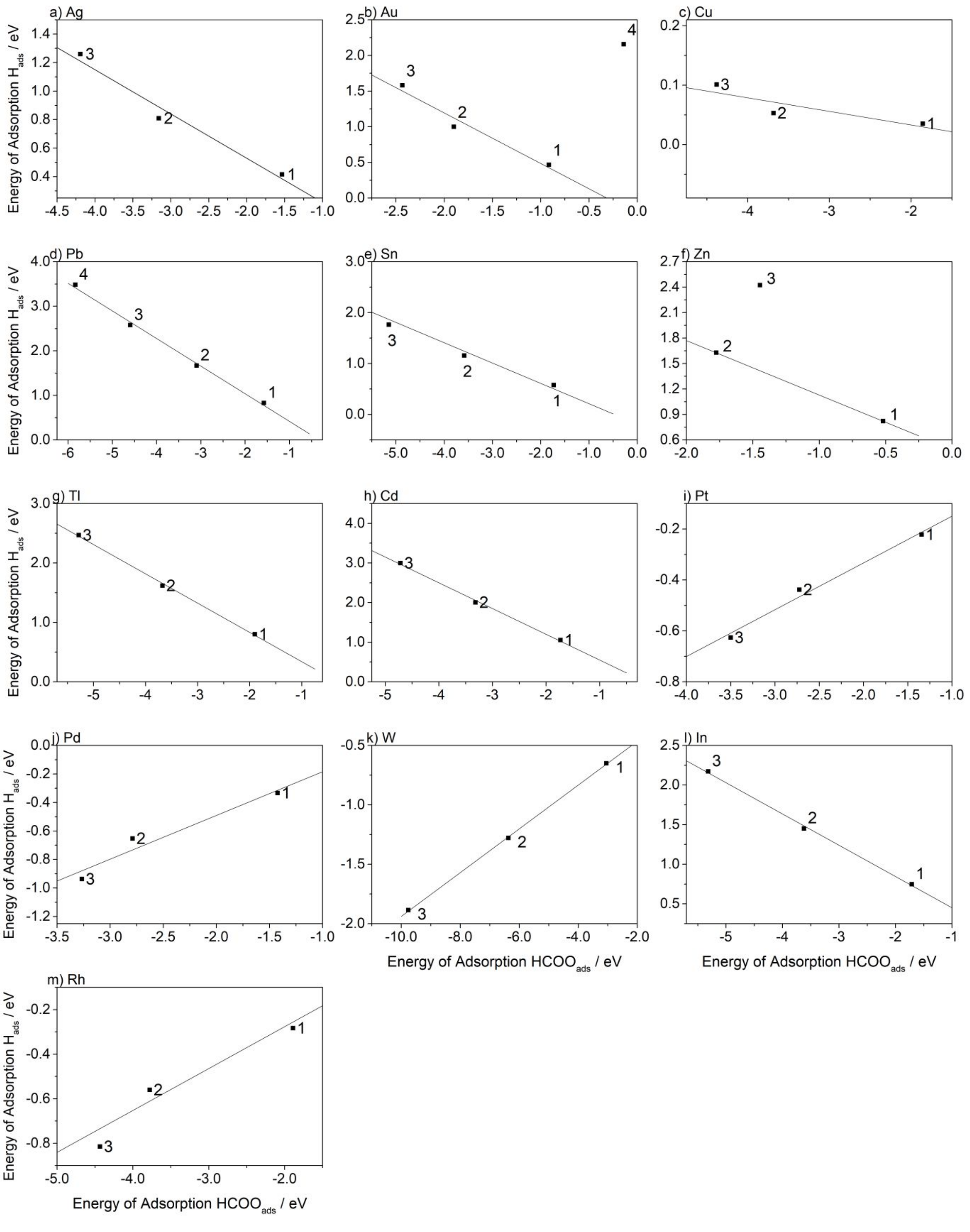


**Figure S4.** All plots of $E_{ads}$ $HCOO_{ads}$ vs. $E_{ads}$ $H_{ads}$ for 1-4 adsorbate of the same type of adsorbate in a 2x3 section of each metal as listed in the figure. The former is the energies of adsorption of 1-4 $CO_2$ adsorbing on the surface through a proton transfer while the latter is the energy of adsorption of a proton. Both are calculated with DFT (see section S1 of the for more details). Each point is labelled with the number of adsorbates (either all $H_{ads}$ or all $HCOO_{ads}$) on a 2x3 surface cell it represents. The lines are plotted from linear regressions of at least 3 of the points – discounting the 4th point if the surface with 4 adsorbates was too packed for a reasonable measurement. The parameters for the fits can be found in the Table 1 of the main article.

## S3 Eliminate Systematic Error

A more detail explanation of why our method can eliminate systematic errors follows. To start, consider that we are calculating pairs of energy values (E1, E2) parametrically from a third variable the surface coverage (θ), (1) and (2).

$$E_1 = F_1(\theta) \quad (1)$$

$$E_2 = F_2(\theta) \quad (2)$$

$$F_1(\theta) = AF_2(\theta) + B \quad (3)$$

Equation (3) is based on the observation that there is a linear relationship between E1 and E2 made in the main text. A is the ratio of energies and what we are interested in calculating. If the values calculated for E1 and E2 have random errors, their effects on the calculation of A by fitting (3) will decrease. However, what we would like to pay attention to is the term B. Calculating A by fitting (3) should completely remove B from the equation. It is particularly interesting because B should be 0 since at θ=0 both energies should be 0:

$$F_1(0) = F_2(0) = 0 \quad (4)$$

And substituting (4) back into (3), shows B to be 0. In the fits calculated from DFT here, (seen in Tables 1,2 and S1 values), B is non-zero. This shows the value of the technique presented here because the results also show there are systematic errors present for it to correct.

## S4 Hydrogen Bonding in $COOH_{ads}$

Hydrogen bonding between $COOH_{ads}$ can be observed in several of the optimized structures generated here, an example of which can be seen in Figure 1 in the main text. The percentage of $COOH_{ads}$ that display hydrogen bonding is relatively constant, but actually increases somewhat for structures with 2 and 3 adsorbates vs those with 1 or 4 adsorbates, as can be seen in figure S5.

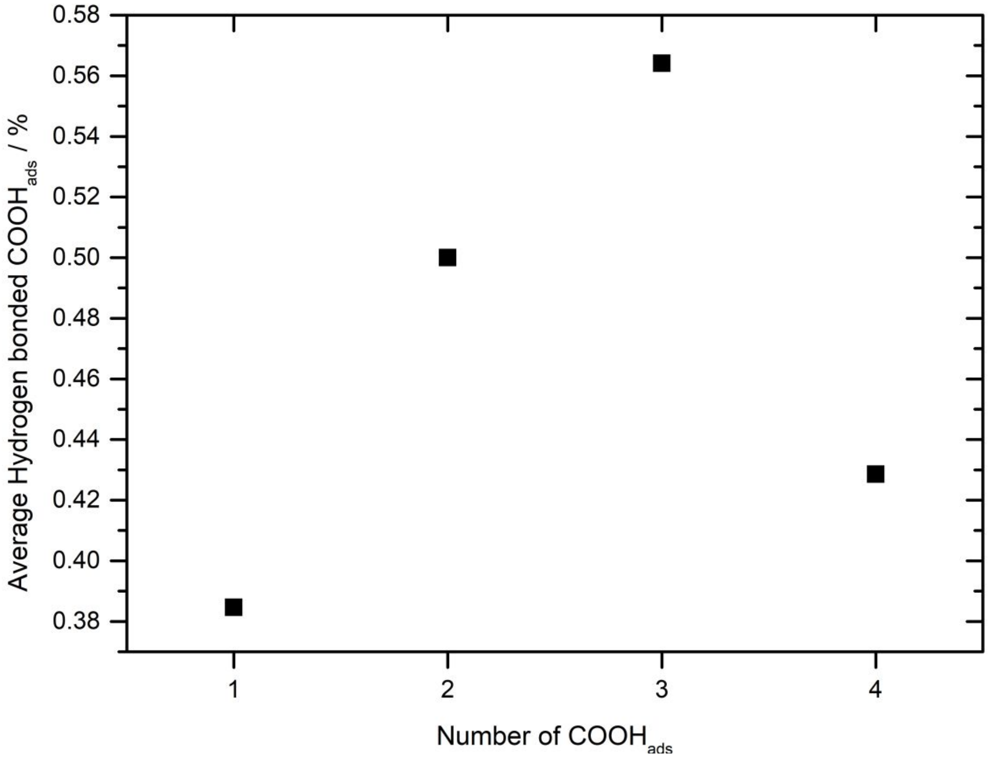


**Figure S5: The percent of $COOH_{ads}$ which show an H on the side structure indicating hydrogen bonding in the minimum energy structure found in this study averaged across the 13 different surfaces for each number of $COOH_{ads}$ in the the 2x3 cell.**

## References


1. Zeradjanin, A. R.; Grote, J.-P.; Polymeros, G.; Mayrhofer, K. J. J., A Critical Review on Hydrogen Evolution Electrocatalysis: Re-Exploring the Volcano-Relationship. *Electroanalysis* **2016**, *28*, 2256-2269.
2. Clark, E. L.; Resasco, J.; Landers, A.; Lin, J.; Chung, L.-T.; Walton, A.; Hahn, C.; Jaramillo, T. F.; Bell, A. T., Standards and Protocols for Data Acquisition and Reporting for Studies of the Electrochemical Reduction of Carbon Dioxide. *ACS Catalysis* **2018**, *8*, 6560-6570.
3. Hori, Y.; Wakebe, H.; Tsukamoto, T.; Koga, O., Electrocatalytic Process of CO Selectivity in Electrochemical Reduction of $CO_2$ at Metal Electrodes in Aqueous Media. *Electrochimica Acta* **1994**, *39*, 1833-1839.
4. Hara, K.; Kudo, A.; Sakata, T., Electrochemical Reduction of Carbon Dioxide under High Pressure on Various Electrodes in an Aqueous Electrolyte. *Journal of Electroanalytical Chemistry* **1995**, *391*, 141-147.
5. Ruban, A.; Hammer, B.; Stoltze, P.; Skriver, H. L.; Nørskov, J. K., Surface Electronic Structure and Reactivity of Transition and Noble Metals1communication Presented at the First Francqui Colloquium, Brussels, 19–20 February 1996.1. *Journal of Molecular Catalysis A: Chemical* **1997**, *115*, 421-429.
6. Santos, E.; Pötting, K.; Schmickler, W., On the Catalysis of the Hydrogen Oxidation. *Faraday Discussions* **2009**, *140*, 209-218.
